# Association between Polycystic Ovary Syndrome, Oral Microbiota and Systemic Antibody Responses

**DOI:** 10.1371/journal.pone.0108074

**Published:** 2014-09-18

**Authors:** Aliye Akcalı, Nagihan Bostanci, Özgün Özçaka, Banu Öztürk-Ceyhan, Pınar Gümüş, Nurcan Buduneli, Georgios N. Belibasakis

**Affiliations:** 1 Institute of Oral Biology, Center of Dental Medicine, University of Zürich, Zürich, Switzerland; 2 Department of Periodontology, School of Dentistry, Ege University, İzmir, Turkey; 3 Department of Endocrinology, School of Medicine, Aydın Government Hospital, Aydın, Turkey; New York University, United States of America

## Abstract

Polycystic ovary syndrome (PCOS) is a hormonal disorder of women that not only is the leading cause of infertility but also shows a reciprocal link with oral health. This study aimed to investigate the hypothesis that the levels of putative periodontal pathogens in saliva and their antibody response in serum are elevated in PCOS, compared to systemic health. A total of 125 women were included in four groups; 45 women with PCOS and healthy periodontium, 35 women with PCOS and gingivitis, 25 systemically and periodontally healthy women, 20 systemically healthy women with gingivitis. Salivary levels of seven putative periodontal pathogens were analyzed by quantitative real-time polymerase chain reaction and serum antibody levels were analyzed by ELISA. In women with PCOS, salivary *Porphyromonas gingivalis*, *Fusobacterium nucleatum, Streptococcus oralis* and *Tannerella forsythia* levels were higher than matched systemically healthy women, particularly in the case of gingivitis. *Aggregatibacter actinomycetemcomitans* and *Treponema denticola* levels were similar among study groups. The presence of PCOS also enhanced *P. gingivalis*, *Prevotella intermedia* and *S. oralis* serum antibody levels, when gingivitis was also present. Gingival inflammation correlated positively with levels of the studied taxa in saliva, particularly in PCOS. The presence of *P. gingivalis* and *F. nucleatum* in saliva also exhibited a strong positive correlation with the corresponding serum antibody levels. In conclusion, as an underlying systemic endocrine condition, PCOS may quantitatively affect the composition of oral microbiota and the raised systemic response to selective members of this microbial community, exerting a confounding role in resultant gingival inflammation and periodontal health. The most consistent effect appeared to be exerted on *P. gingivalis*.

## Introduction

Polycystic ovary syndrome (PCOS) is the most common endocrine disorder, affecting women of reproductive age with the prevalence ranging from 6.5% to 8% according to the National Institutes of Health (NIH) criteria [1]. PCOS is considered to be a metabolic syndrome with cardiovascular [2], insulin-dependent diabetes, dyslipidemia and endothelial dysfunction [3] and visceral obesity [4] risk factors. PCOS is characterised by chronic low-grade inflammation [5] and it is likely to be responsible for metabolic abnormalities. Recently, it was reported that certain pro-inflammatory cytokines, such as interleukin-6 (IL-6), interleukin-17 (IL-17), tumor necrosis factor-α (TNF-α) were elevated in women with PCOS, compared to systemically healthy individuals [6], [7]. Prolonged low-grade inflammatory state can be caused by chronic infections such as gingivitis, which is a common pathology seen in patients with PCOS [8]. The primary etiological factor for periodontal diseases is microorganisms in the dental plaque, such as *Porphyromonas gingivalis, Treponema denticola, Tannerella forsythia, Aggregatibacter actinomycetemcomitans* and *Prevotella intermedia* species, which can also be detected in saliva [9]. Importantly, the effect of female steroid hormones on the composition of oral microbiota has been reported in puberty, menstruation, pregnancy and with oral contraceptive usage [10]. Nevertheless, there is still limited information about the composition of oral microbiota, with regards to systemic inflammatory conditions triggered by hormonal disorders, such as PCOS. Taking into consideration that periodontal diseases are chronic infections that cause a low-grade chronic systemic inflammation [11] it is plausible to consider an association with hormonal disorders, such as PCOS.

The oral microbiota may trigger systemic antibody responses in patients with periodontal disease [12], [13], [14]. It was previously shown that patients with chronic or aggressive periodontitis have higher serum anti-bacterial IgG antibodies compared to periodontally healthy individuals with no clinical signs of early-onset periodontitis [15], [16]. However, serum antibody responses to periodontal pathogens neither confer immunity against periodontal disease [17], nor are they considered as an auxiliary measure for the diagnosis of this disease [18]. To the contrary there is evidence that the severity of periodontal disease may negatively correlate with local and systemic antibody titers to periodontal pathogens, such as *P. gingivalis* and *A. actinomycetemcomitans* [19]. The carriage of these two species alone may be a determinant for their systemic antibody response, but not for the periodontal health status [16]. On the other hand, the clinical definition of the periodontitis may be a crucial factor that can modify the association between *P. gingivalis* serum antibody titers and the disease [20]. Because conspicuous differences exist in antibody titers to periodontal pathogens between periodontal health and disease, even after successful periodontal therapy, the systemic antibody responses may mark the history of past periodontal infection [21]. It needs to be further investigated if underlying systemic conditions can modify the serum antibody responses to periodontal pathogens, as well as their relationship is to periodontal inflammation.

To date, the relationship between oral microbiota, gingival inflammation and systemic antibody response in presence of PCOS has not been investigated. The hypothesis of this study is that salivary levels of putative periodontal pathogens, as well as the serum antibody levels to them are elevated in patients with PCOS, particularly in the presence of gingival inflammation. Therefore, the aim of the study was to investigate the levels of seven oral taxa, including *P. gingivalis*, *T. denticola*, *T. forsythia, A. actinomycetemcomitans, P. intermedia*, *F. nucleatum* and *S. oralis*, and the associated serum antibody responses in women with PCOS, particularly in presence of gingivitis.

## Materials and Methods

### Study population and clinical examination

Forty-five women with PCOS and healthy periodontium, 35 women with PCOS and gingivitis, 25 systemically and periodontally healthy women, and 20 systemically healthy women with gingivitis were recruited in this study from the outpatient clinic of Department of Endocrinology, School of Medicine, Aydın Government Hospital, Turkey, between October 2012 and April 2013. The study was conducted in full accordance with ethical principles, including the World Medical Association’s Declaration of Helsinki, as revised in 2008 and was approved by the Ethics Committee of the Ege University with the protocol number 13-5.1/13. The study protocol was explained and written informed consent was received from each individual prior to enrolment. The study conforms to STROBE guidelines for observational studies [22]. Complete medical and dental histories were obtained from each individual. Inclusion to the PCOS group was based on the criteria by Rotterdam with the presence of at least two of the following: polycystic ovaries, oligomenorrhea and/or anovulation, hyperandrogenism (clinical and/or biochemical) [23]. Inclusion criteria for the systemically healthy group were; history of regular menstrual cycles, no clinical or biochemical sign of hyperandrogenism, matching with the PCOS group in body mass index (BMI) and exclusion of PCOS by ultrasound examination. Systemic exclusion criteria were hyperandrogenism (tested by levels of 17-α-hydroxyprogesterone), diabetes mellitus, hyperprolactemia, congenital adrenal hyperplasia, thyroid disorders, Cushing’s syndrome, hypertension, hepatic or renal dysfunction, BMI>30 kg/m^2^, and cardiovascular diseases. None of the patients had been using medications such as oral contraceptive agents, steroid hormones, insulin-sensitizing drugs and antibiotics or anti-inflammatory drugs that could affect their periodontal status within the last 6 months before inclusion in the study. Upon confirmation of eligibility for enrolment in the study, all individuals returned to the clinic for clinical periodontal measurements including probing depth (PD), plaque index (PI) [24], and bleeding on probing (BOP; +/−). Clinical periodontal measurements were performed at 6 sites on each tooth (mesio-buccal, mid-buccal, disto-buccal, mesio-lingual, mid-lingual and disto-lingual locations), except for third molars, using a Williams periodontal probe (Hu-Friedy, Chicago, IL). BOP was deemed positive if it occurred within 15 seconds after application of the probe. The clinical periodontal measurements were performed by a single calibrated examiner (Aliye Akcalı).

Patient selection was based on clinical and radiographic criteria proposed by the 1999 International World Workshop for a Classification of Periodontal Disease and Conditions [25]. Diagnosis of gingivitis was assigned when BOP scores >50% of all sites, PD <3 mm at 90% of the measured sites and no more than one site had a PD >4 mm or clinical attachment level (CAL) ≤1 mm, and no clinical and/or radiographic sign of periodontitis was evident.

### Collection and processing of saliva and serum

All salivary samples were collected in the morning between 8∶00 am and 9∶00 am. Participants were asked to avoid oral hygiene measures (flossing, brushing and mouth-rinses), eating, and drinking for 2 h before collection. For saliva collection, the individuals were asked to rinse their mouth with distilled water for 2 min and wait for 10 min after rinsing before expectorating whole saliva into sterile 50 ml tubes for 5 min. The collected saliva samples were then placed on ice, supplemented with EDTA-free Protease Inhibitor Cocktail (Roche Applied Science, Switzerland) prior to centrifuging at 10000×g for 15 min at 4°C. The resulting supernatants were then immediately aliquoted and frozen at −80°C for future use, whereas the resulting pellet was re-suspended in 500 µl of phosphate-buffered saline (PBS), and used for DNA extraction, as described further.

For serum collection, a total of 5 ml of venous blood were taken into BD Vacutainer blood collection tubes with silicone-coated interior (BD Diagnostics, Franklin Lakes, NJ, USA) by a standard venipuncture method. The collected serum samples were left at room temperature to allow for blood clotting and then centrifuged at 1500×g for 15 min at 4°C to remove the fibrin clot and cellular elements. All serum samples were then immediately aliquoted and frozen at −80°C.

### Bacterial quantification by quantitative real-time Polymerase Chain Reaction (qPCR)

For determining microbiological composition of saliva, qPCR was employed in order to define the presence and levels of seven different oral taxa in the salivary samples. A volume of 300 µl of the re-suspended pellet (described above) was used for DNA extraction using the GenElute bacterial genomic DNA kit (Sigma Aldrich, Buch, Switzerland) according to the manufacturer’s guidelines for the Gram positive lysis protocol, with the following modifications. Mutanolysin (Sigma Aldrich, Buchs, Switzerland) was added for the extraction of streptococcal DNA from the samples. The lysis steps were expanded from 30 min to 1 h (lysozyme/mutanolysin step), and from 10 min to 30 min (proteinase K step), respectively. The recommended RNase A treatment step for 2 min was also performed, in-between the two lysis steps.

Following extraction, the DNA concentration was determined using a NanoDrop ND-1000 (Thermo-Fisher Scientific, Wohlen, Switzerland). All species-specific primers for *P. gingivalis*, *T. denticola*, *T. forsythia, A. actinomycetemcomitans, P. intermedia*, *F. nucleatum* and *S. oralis* used in the qPCR reaction were described recently [26], [27]. For determination of total bacterial counts, universal primers were used as described elsewhere [28]. DNA concentration was diluted to 20 ng/reaction. The qPCR reaction was run in a total volume of 15 µl, containing 7.5 µl of 2x SYBR Green PCR Master Mix (Life Technologies, Zug, Switzerland), 6 µl of DNA template and 1.5 µl of primer pair solution (1 µM/reaction). Amplification of the extracted DNA template was performed in a real time PCR system (Step One Plus, Applied Biosystems, Life Technologies, Basel, Switzerland by a initial incubation of 10 min at 95°C, followed by 40 cycles of 15 s at 95°C and 1 min at 60°C). From the obtained Cq values, number of copies/reaction was calculated for each sample according to the standard curve values obtained from the amplification of known DNA amounts extracted from pure culture isolates of each bacterial taxa (lowest amount: 0.0001 ng/reaction). The theoretical bacterial numbers in each sample were calculated as total bacterial DNA counts based on the measured amount of DNA and the estimated genome weight [26], [27].

### Immunological response assessment by enzyme-linked immunosorbant assay (ELISA)

For determining the systemic antibody responses to oral microbiota, ELISA was employed in order to define presence and levels of the antibody levels of the designated seven different oral taxa, in 124 serum samples (PCOS with healthy periodontium, n = 45; PCOS with gingivitis, n = 35; systemically and periodontally healthy, n = 24; systemically healthy with gingivitis, n = 20). The following strains were used as bacterial antigens: *P. gingivalis* ATCC 33277T (OMZ 308), *T. denticola* ATCC 35405 (OMZ 661), *T. forsythia* (OMZ 1047), *A. actinomycetemcomitans* ATCC 33384 (OMZ 295), *P. intermedia* (OMZ 278), *F. nucleatum* (OMZ 598), and *S. oralis* (OMZ 607). Once the bacterial cultures were grown, then the broth was removed by centrifugation at 4000×g, the bacterial cell pellets were washed three times with PBS. The density of bacterial suspension was adjusted to an optical density of OD_550 nm_ 0.10 in PBS with 0.02% sodium azide. For antigen coating, the ELISA microplates were incubated with 200 µl of each bacterial culture and fixed with 0.1% glutaraldehyde solution at room temperature for 45 min. Afterwards, the unspecific binding was blocked by 1% bovine serum albumin (BSA) for 2 h at 37°C in borate buffer saline (BBS) (137 mM H_3_BO_3_, 25 mM Na_2_B_4_O_7_, 75 mM NaCl, pH = 8), and then the wells were washed twice with BBS.

The reference serum with relatively high titer was selected as described earlier [29]. Two-fold serial dilutions were made with use of the BBS buffer. The serially diluted reference serum samples as well as the diluted test serum samples (predetermined optimal dilutions for each bacteria were 1∶100 for all other species except for *T. denticola* which was 1∶10) were added to each well, and incubated for 1 h at 37°C. Additionally, the last two wells were blank (without any samples) and served as negative controls. Each well was washed five times with BBS, and incubated with 100 µl of horseradish peroxidase (HRP)-conjugated goat anti- human IgG (H+L) antibodies at 1∶5000 dilution (GAHu/IgG(Fc)/PO, 6.3 mg/ml, Nordic Immunological Laboratories, Netherlands) for 2 h at 37°C. Thereafter, colour development was performed with 100 µl of substrate buffer for 15 min at room temperature, and stopped by addition of 50 µl of 2.5 M H_2_SO_4._ The absorbance was measured spectrophotometrically at 492 nm using a microplate reader (Epoch, BioTek, Luzern, Switzerland). The reciprocal relation between the reference serum dilution (assigned as arbitrary ELISA unit; EU) and OD_492_ measurement was approximated by a 4-parameter polynomial curve. Highest mean absorbance of the reference serum (1∶400 dilution) was assigned to 100 ELISA Units (EU). The arbitrary units of the test samples were then determined by comparing the absorbance of the samples to the standard curve.

### Statistical analysis

The distribution of the data was evaluated by D’Agostino-Pearson omnibus normality test. Comparisons between all groups for non-normally distributed variables (bacterial counts and antibody levels) were performed by the Kruskal-Wallis test and Dunn’s test was used in order to correct for multiple comparisons. For normally distributed variables (age, PI, BOP and PD) ordinary two-way ANOVA test with Holm-Sidak’s multiple comparison test (family-wise significance and confidence level 0.05) was used. Correlations between BOP, PI scores, PD levels, numeric levels of detection of different taxa in saliva and antibody levels of different taxa in serum according to systemic health status, were analysed by Spearman's correlation test. For this, in order to control the false discovery rate in multiple testing, Benjamini and Hochberg method was applied. The Statistical analyses were conducted using the statistical software (GraphPad Prism version 6.00c for Mac OS X, GraphPad Software, La Jolla California USA), and statistical significance was considered at *p*<0.05.

## Results

### Clinical findings

Demographics and full mouth clinical findings are provided in Table 1. Mean PD and CAL were below 3 mm in all the study groups, whereas PI, BOP and PD scores were significantly higher in the gingivitis groups, either systemically healthy or with PCOS. Clinical periodontal parameters were similar in the gingivitis groups with or without PCOS.

**Table 1:** Age demographics and clinical periodontal measurements.

	PCOS - health (n = 45)	PCOS - gingivitis (n = 35)	Health - health (n = 25)	Health - gingivitis (n = 20)
Age (Years)	25.02±5.86	26.40±5.77	25.84±4.07	26.40±5.42
PD (mm)	1.32±0.39	1.95±0.24****	1.26±0.45	1.94±0.22****
PI (Score 0–3)	0.75±0.34	2.13±0.39****	0.82±0.46	2.05±0.43********
BOP (%)	8.80±10.73	73.45±17.31****	10.21±13.82	68.52±21.73****

****p<0.0001, PD = Probing pocket depth (mm), PI = Plaque Index, BOP = Bleeding on probing (%). Clinical diagnosis groups: PCOS with periodontal health (PCOS - health); PCOS with gingivitis (PCOS - gingivitis); systemically healthy with periodontal health (Health - health); systemically healthy with gingivitis (Health - gingivitis). Values are shown as mean ± standard deviation.

### Analysis of bacterial counts in saliva

**Figure 1:**
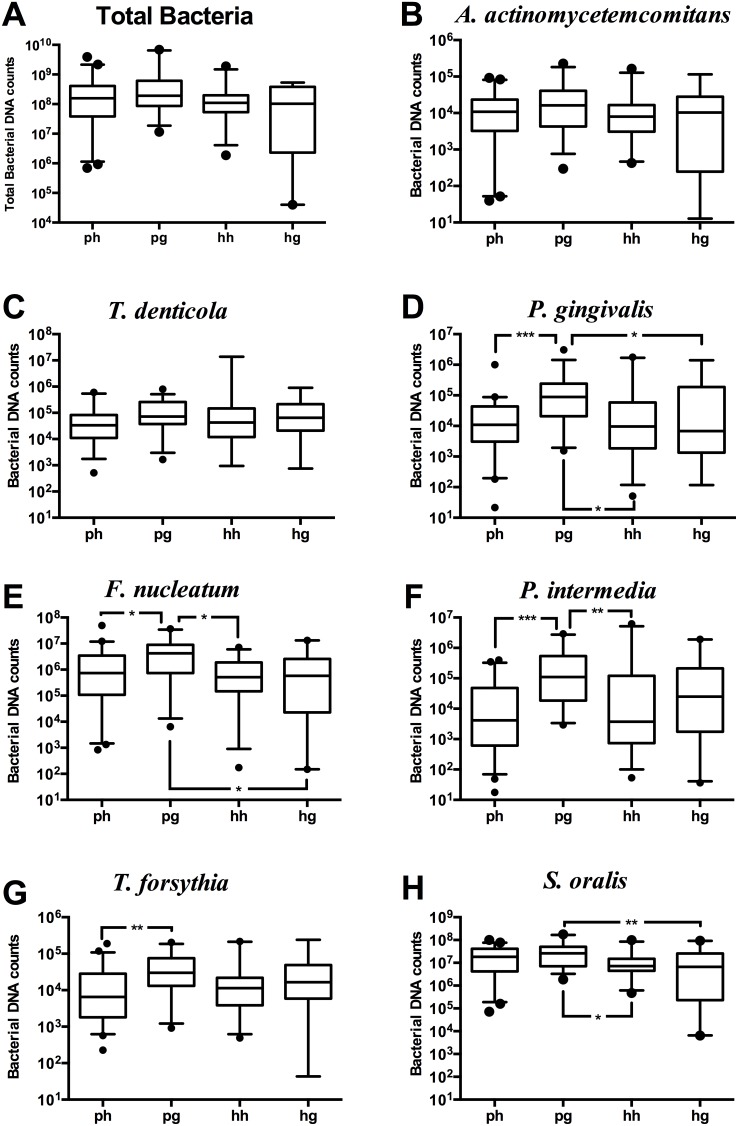
Levels of detection of different species in saliva. Levels of detection of different species in saliva from PCOS with periodontal health (ph) (n = 45); PCOS with gingivitis (pg) (n = 35); systemically healthy with periodontal health (hh) (n = 25); systemically healthy with gingivitis (hg) (n = 20). The individual values represent bacterial counts in saliva (A); Total bacterial counts (B); *A. actinomycetemcomitans*, (C); *T. denticola*, (D); *P. gingivalis*, (E); *F. nucleatum*, (F); *P. intermedia*, (G); *T. forsythia*, and (H); *S. oralis* in both groups. The horizontal lines in the boxplots represent the median values and the whiskers represent the 5–95 percentiles. Values below and above the whiskers are drawn as individual dots. *p<0.05, **p<0.01, ***p<0.001.

The microbiological composition of saliva was evaluated using qPCR either for total bacterial counts, or for each one of the seven individual species. Their frequency of detection was also calculated (Table 2). Total bacterial counts were statistically similar between the four study groups (Figure 1A). Among the specific species, no differences were found in the numbers of *A. actinomycetemcomitans* (Figure 1B) and *T. denticola* (Figure 1C) between clinical groups. The levels of *P. gingivalis* (Figure 1D) and *F. nucleatum* (Figure 1E) were also found to be similar in systemically healthy women, irrespective of the presence of gingivitis. In contrast, these were significantly higher in women with PCOS and gingivitis, than any of the other three groups. Accordingly, *P. intermedia* levels were also significantly higher in women with PCOS and gingivitis, compared to systemically or periodontally healthy women, but there was no difference between the two gingivitis groups (Figure 1F). *T. forsythia* levels were higher in women with PCOS and gingivitis compared to periodontal health, but there were no differences between any of the other clinical group comparisons (Figure 1G). *S. oralis* was significantly higher in women with PCOS and gingivitis, than systemically or periodontally healthy women (Figure 1H).

**Table 2:** Frequency of detection of different species per clinical group.

Species	PCOS - health	PCOS - gingivitis	Health - health	Health - gingivitis
*P. gingivalis*	45/45 (100%)	34/35 (97%)	25/25 (100%)	18/20 (90%)
*T. denticola*	32/45 (71%)	34/35 (97%)	18/25 (72%)	17/20 (85%)
*T. forsythia*	42/45 (93%)	35/35 (100%)	23/25 (92%)	19/20 (95%)
*A. actinomycetemcomitans*	44/45 (98%)	35/35 (100%)	25/25 (100%)	18/20 (90%)
*P. intermedia*	43/45 (96%)	33/35 (94%)	25/25 (100%)	20/20 (100%)
*F. nucleatum*	45/45 (100%)	35/35 (100%)	25/25 (100%)	19/20 (95%)
*S. oralis*	45/45 (100%)	35/35 (100%)	25/25 (100%)	20/20 (95%)

Clinical diagnosis groups: PCOS with periodontal health (PCOS - health); PCOS with gingivitis (PCOS - gingivitis); systemically healthy with periodontal health (Health - health); systemically healthy with gingivitis (Health - gingivitis).

### Analysis of bacterial antibody levels in serum

**Figure 2:**
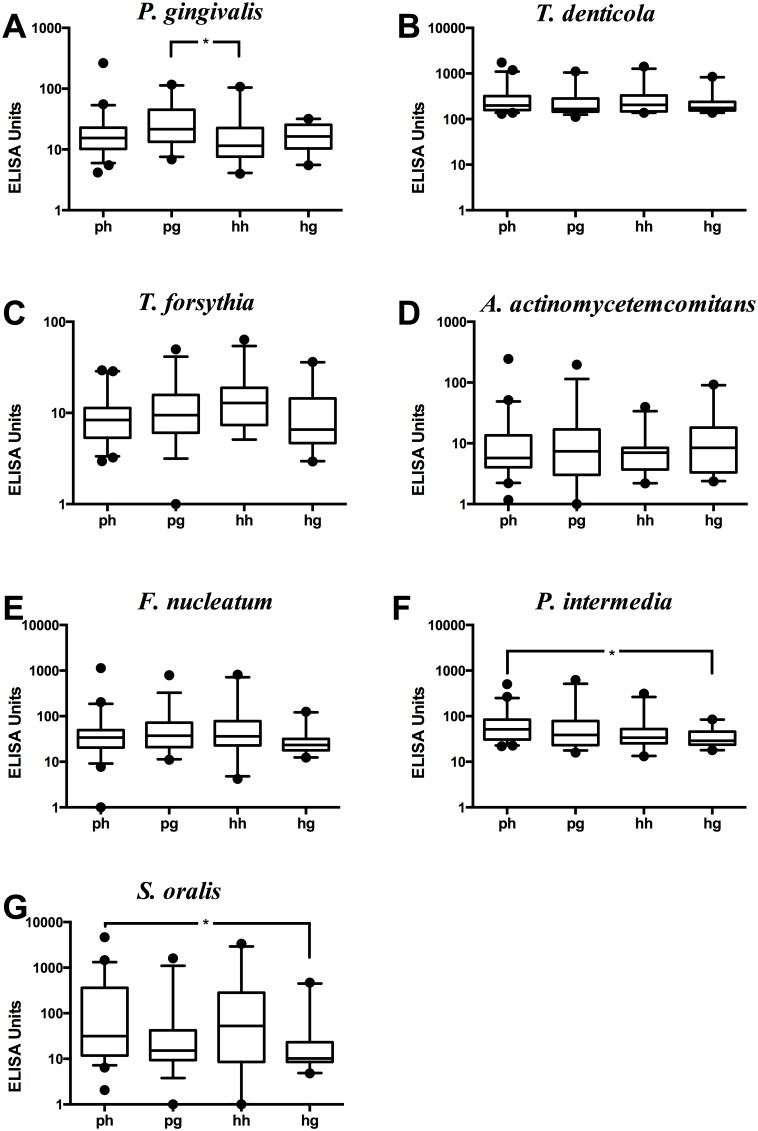
Antibody levels of different species in serum. Antibody levels of different species in serum from PCOS with periodontal health (ph) (n = 45); PCOS with gingivitis (pg) (n = 35); systemically healthy with periodontal health (hh) (n = 24); systemically healthy with gingivitis (hg) (n = 20). The individual values represent antibody levels in serum (A); *P. gingivalis*, (B); *T. denticola*, (C); *T. forsythia*, (D); *A. actinomycetemcomitans*, (E); *F. nucleatum*, (F); *P. intermedia*, and (G); *S. oralis* in both groups. The horizontal line indicates the study groups. The horizontal lines in the boxplots represent the median values and the whiskers represent the 5–95 percentiles. Values below and above the whiskers are drawn as individual dots. *p = 0.02, **p = 0.01.

Serum antibody responses to the selected oral species were further investigated. No pronounced differences were found between clinical groups. A statistically significant difference was observed in the case of antibody responses to *P. gingivalis*, but only between women with PCOS and gingivitis, compared to systemically and periodontally healthy women (Figure 2A). No differences between any of the clinical groups were found for *T. denticola* (Figure 2B), *T. forsythia* (Figure 2C), *A. actinomycetemcomitans* (Figure 2D) and *F. nucleatum* (Figure 2E). Another observation was that *P. intermedia* and *S. oralis* antibody levels were significantly higher in the periodontally healthy women with PCOS, compared to the systemically healthy women with gingivitis (Figure 2F and 2G, respectively).

### Correlation analyses between clinical, microbiological and serological parameters

The Spearman’s rank correlation analysis was used to investigate the correlation between clinical parameters, levels of detection of different species in saliva (Table 3) and antibody levels in serum (Table 4) according to the systemic health status (PCOS or systemically healthy). Following the calculation of exact *p* values, Benjamini and Hochberg method was applied to identify the corrected significance level, and values below the threshold level were accepted statistically significant. In the systemically healthy group, a significant positive correlation was indicated between BOP and levels of *P. intermedia*, *T. forsythia* and *T. denticola* in saliva, while *P. gingivalis,* which was at the border of being significant (*p* = 0.051). In case of the PCOS group, the correlations between BOP and all seven different species were significant. Importantly, all these correlations proved to be stronger within the PCOS group, rather than the systemically healthy group (Table 3).

**Table 3:** Correlation between clinical periodontal parameters and salivary bacteria counts.

		PCOS							Systemically Healthy						
		Pg	Td	Tf	Aa	Pi	Fn	Sora	Pg	Td	Tf	Aa	Pi	Fn	Sora
PD	r	0.447	0.424	0.438	0.180	0.396	0.266	0.286	0.174	0.195	0.288	0.023	0.340	0.177	0.117
	*p*	<0.001*	<0.001*	<0.001*	0.110	<0.001*	0.017*	0.010*	0.253	0.198	0.055	0.882	0.022	0.244	0.444
BOP%	r	0.549	0.562	0.541	0.288	0.470	0.404	0.305	0.293	0.438	0.454	0.130	0.479	0.279	0.196
	*p*	<0.001*	<0.001*	<0.001*	0.010*	<0.001*	<0.001*	0.006*	0.051	0.003*	0.002*	0.393	0.001*	0.063	0.198
PI	r	0.587	0.610	0.580	0.301	0.465	0.398	0.350	0.297	0.407	0.403	0.112	0.429	0.144	0.113
	*p*	0.001*	<0.001*	<0.001*	0.007*	<0.001*	<0.001*	0.001*	0.048	0.006*	0.006*	0.465	0.003*	0.346	0.458

Species*: P. gingivalis* (Pg); *T. denticola* (Td); *T. forsythia* (Tf); *A. actinomycetemcomitans* (Aa); *P. intermedia* (Pi); *F. nucleatum* (Fn); *S. oralis* (Sora).

*Significant correlations according to Benjamini and Hochberg corrected significance level.

**Table 4:** Correlation between clinical periodontal parameters and serum antibody levels against oral bacteria.

		PCOS							Systemically Healthy						
		Pg	Td	Tf	Aa	Pi	Fn	Sora	Pg	Td	Tf	Aa	Pi	Fn	Sora
PD	r	0.081	−0.080	0.172	−0.042	−0.124	−0.021	−0.035	0.061	0.030	−0.227	−0.088	−0.045	−0.170	−0.300
	*p*	0.475	0.483	0.128	0.708	0.272	0.852	0.760	0.695	0.845	0.139	0.571	0.769	0.270	0.048
BOP%	r	0.251	−0.092	0.158	0.003	−0.114	0.037	−0.091	0.255	−0.035	−0.415	0.013	−0.062	−0.279	−0.434
	*p*	0.025	0.419	0.161	0.977	0.313	0.746	0.421	0.095	0.824	0.005*	0.935	0.688	0.067	0.003*
PI	r	0.245	−0.038	0.155	0.015	−0.108	0.020	−0.057	0.253	−0.023	−0.262	0.002	−0.029	−0.187	−0.403
	*p*	0.029	0.738	0.169	0.897	0.343	0.860	0.618	0.098	0.883	0.086	0.992	0.852	0.224	0.007*

Species*: P. gingivalis* (Pg); *T. denticola* (Td); *T. forsythia* (Tf); *A. actinomycetemcomitans* (Aa); *P. intermedia* (Pi); *F. nucleatum* (Fn); *S. oralis* (Sora).

*Significant correlations according to Benjamini and Hochberg corrected significance level.

In terms of serum antibody responses, a significantly negative correlation was found between BOP and *T. forsythia* or *S. oralis* in the systemically healthy group (Table 4). When the association between bacterial salivary levels and their serum antibody levels were investigated, a significant positive correlation was found in the cases of *P. gingivalis* and *F. nucleatum* (Table 5).

**Table 5:** Correlation between salivary bacteria counts and serum antibody levels.

			Salivary bacteria counts						
			Pg	Td	Tf	Aa	Pi	Fn	Sora
	Pg	r	0.306	0.211	0.168	0.036	0.059	0.131	−0.032
		*p*	0.001*	0.019	0.062	0.694	0.516	0.146	0.728
	Td	r	−0.088	−0.120	−0.144	0.064	−0.157	−0.052	−0.009
		*p*	0.330	0.184	0.111	0.483	0.081	0.568	0.923
	Tf	r	−0.008	−0.023	−0.010	0.145	−0.130	0.074	0.043
		*p*	0.933	0.797	0.910	0.107	0.150	0.416	0.636
Serum	Aa	r	−0.064	0.074	0.057	0.121	−0.034	0.001	−0.002
antibody		*p*	0.477	0.416	0.532	0.182	0.708	0.990	0.980
levels	Pi	r	0.113	−0.029	−0.074	0.113	−0.064	0.078	0.021
		*p*	0.210	0.753	0.411	0.213	0.478	0.392	0.814
	Fn	r	0.161	−0.052	−0.044	0.128	0.054	0.239	0.134
		*p*	0.074	0.563	0.627	0.157	0.549	0.007*	0.137
	Sora	r	−0.030	−0.114	−0.134	0.165	−0.040	0.167	0.103
		*p*	0.744	0.208	0.138	0.067	0.660	0.063	0.255

Species*: P. gingivalis* (Pg); *T. denticola* (Td); *T. forsythia* (Tf); *A. actinomycetemcomitans* (Aa); *P. intermedia* (Pi); *F. nucleatum* (Fn); *S. oralis* (Sora).

*Significant correlations according to Benjamini and Hochberg corrected significance level.

## Discussion

The incidence and clinical presentation of plaque-induced gingivitis are affected by increased sex steroid hormone levels [10]. Puberty has a transient effect on the inflammatory status of the gingiva, but the severity or the time of onset of gingival inflammation varies in different studies [30]. Severity of gingivitis during the pregnancy can be increased independently from dental plaque accumulation [31]. A quantitative shift in the subgingival levels of Gram-negative anaerobic microorganisms occurs during the second trimester of pregnancy [32], [33]. The likely explanation is that the local accumulation of active progesterone and oestrogen may provide the essential nutrients that selectively enhance their growth [34], [35]. Androgen production is a major trait of PCOS and essential for follicle development [36]. Although the overproduction of luteinizing hormone is evident, the absence of the peak level of the hormone results in higher levels of progesterone and estrogen production [37]. Such hormonal changes in PCOS are likely to influence the salivary levels of putative periodontal pathogens, or their systemic antibody responses, particularly when associated with gingival inflammation.

To our knowledge, this is the first study to investigate the association between PCOS and oral microbiota in saliva and their serum antibody responses, in regards to gingival inflammation. The findings demonstrated that the levels of most of the studied putative periodontal pathogens, except *A. actinomycetemcomitans* and *T. denticola*, were elevated in women with PCOS and gingivitis compared to the matched periodontally healthy controls. PCOS appeared to have an enhancing effect on the levels of *P. gingivalis* and *F. nucleatum* and their association with gingival inflammation, hinting towards a microbial specificity.

Measurement of serum antibody levels provides information on host reaction to the oral microbiota [20], as they may be associated with the progression from periodontal health to disease [38], or define disease-susceptible or disease-resistant individuals [39]. In systemically healthy individuals there were no major differences in serum antibody levels according to periodontal status, despite differences in the subgingival microbiota [40] However the systemic health status may alter the serum antibody responses to periodontal pathogens, as demonstrated in patients with poor glycaemic control [41]. To this extent, serum antibody levels to *C. rectus* were elevated in type 2 diabetes, whereas antibody levels to *P. gingivalis* were elevated in periodontitis, irrespective of diabetic health status [42]. Recent evidence also shows that serum antibody levels of *A. actinomycetemcomitans, P. gingivalis* and *P. intermedia* are associated with an increased risk of coronary heart disease [43]. In the present study, serum antibody levels to *P. gingivalis*, *P. intermedia* and *S. oralis* were elevated in the presence of PCOS. This may indicate that this endocrine disorder may affect the antigenic susceptibility to these species. Although in systemic health no positive correlation was found between gingival inflammation (as evaluated by BOP scores) and serum antibody levels to any of the studied species, in the presence of PCOS a positive correlation was found only for *P. gingivalis*. Moreover, salivary counts and serum antibody levels of *P. gingivalis*, as well as *F. nucleatum*, strongly correlated with each other.

Serum antibody levels may provide insights for understanding individual systemic host responses to periodontal microbiota, although the particular associations are not well understood to date. To this extent, a national US survey has shown that demographic, behavioural, oral and general health-related characteristics were strong determinants of systemic antibody responses to periodontal bacteria [20], [44]. In this regard, it is reasonable to consider that PCOS may well be a modifier of periodontal health status and systemic antibody responses in the female population.

In conclusion, PCOS may affect the composition of the oral microflora and modify the systemic antibody responses to selective members of this microbial community, with repercussions in periodontal health. Among the studied taxa, the strongest effect was exerted on *P. gingivalis*, as PCOS enhances its presence in saliva and the serum antibody responses to it, particularly in the presence of gingival inflammation. Hence, this study reveals that the positive association between oral microbiota and gingival inflammation is further potentiated by this endocrine disorder.
